# Neurocircuitry and Neuroanatomy in Panic Disorder: A Systematic Review

**DOI:** 10.31083/AP38756

**Published:** 2025-02-28

**Authors:** Peter Kyriakoulis, Clarissa Wijaya, Laiana Quagliato, Rafael C. Freire, Antonio E. Nardi

**Affiliations:** ^1^School of Psychology, Faculty of Arts, Health and Design, Swinburne University of Technology, Melbourne, VIC 3122, Australia; ^2^Positive Psychology Centre, Clinical Research, Melbourne, VIC 3166, Australia; ^3^Laboratory of Panic and Respiration, Institute of Psychiatry, Federal University of Rio de Janeiro, 21941-617 Rio de Janeiro, Brazil; ^4^Department of Psychiatry and Centre for Neuroscience Studies, Queen’s University, Kingston, ON K7L 3N6, Canada

**Keywords:** panic disorder, neurocircuitry, neuroanatomy, etiology, systematic review

## Abstract

**Background:**

This review updates our understanding of the neuroanatomical and neurocircuitry factors involved in panic disorder (PD). Many aspects remain undetermined.

**Methods:**

Clinical studies and a randomized controlled trial were identified via PubMed database and included in this review.

**Results:**

The search, following PRISMA guidelines, identified 13 human studies and 3 animal studies. Nine human studies compared brain activity and connectivity between regions in PD patients. Neural activity in the amygdala was highlighted in six studies. The hippocampus had higher activation in PD patients compared to those with social phobia, but generally showed less activity compared to healthy controls. The parahippocampal gyrus and thalamus exhibited greater activation in PD patients than healthy controls. Activity in the prefrontal cortices was also noted, particularly the ventromedial prefrontal cortex (vmPFC), ventrolateral prefrontal cortex (vlPFC), dorsomedial prefrontal cortex (dmPFC), and dorsolateral prefrontal cortex (dlPFC). Other regions involved included the dorsal midbrain, left brainstem (showing hyperactivation), S1, and right caudate, which showed increased activity in PD patients. The left intraparietal sulcus (IPS) exhibited hypoactivation in response to predictable cues compared to unpredictable or neutral cues within the default mode network (DMN). Three animal studies suggested that electrical and chemical activation of the dorsal periaqueductal gray (dPAG) in rats elicited fight-or-flight behaviors, providing a model for panic attacks.

**Conclusions:**

Neuroimaging studies suggest several key regions involved in PD pathophysiology, including the brainstem, amygdala, hippocampus, parahippocampal gyrus, thalamus, insula, and prefrontal and cingulate cortices. Hypersensitivity in the brainstem and amygdala plays a role in activating the fear network. Further prospective studies are needed to identify the neuroanatomical sites involved in PD and fear circuitry.

## Main Points

1. Neural activity in the amygdala is highlighted in panic disorder (PD) patients.

2. The hippocampus, left-brain stem, and cingulate cortices were found to have significantly higher activation in PD.

3. PD patients were found to have greater activation in the parahippocampal gyrus and thalamus compared with healthy controls.

4. Multiple prefrontal cortices were implicated in the neural activity of PD patients, including the ventromedial prefrontal cortex, ventrolateral prefrontal cortex, dorsomedial prefrontal cortex, and dorsolateral prefrontal cortex.

5. The dorsal midbrain, left brainstem, S1, and right caudate were found to be hyperactivated in PD patients.

## 1. Introduction

Panic disorder (PD) is a severe anxiety disorder characterized by a high degree of distress that is often occupationally and socially disabling [1, 2]. PD is defined by spontaneous and recurrent panic attacks (PAs) [3], likely initiated by complex fear circuitry in the brain and which remains poorly understood [4].

The fear circuitry comprises the amygdala, thalamus, hippocampus, insula, and prefrontal cortex, and involves neurobiological fear responses including neurochemical, neuroendocrine, and behavioral responses adaptive to survival [5]. Several neuroanatomical models have been proposed to explain panic and to investigate the fear circuits involved in the brain [6, 7, 8].

The primary objective of this review is to identify which neuroanatomical areas are implicated in the pathophysiology and etiology of PD. The secondary objective is to identify sophisticated translational models to evaluate how animal research enhances our understanding of the neurobiological foundations and pathophysiology of PD in humans. This systematic review attempts to update and consolidate the knowledge of PD in neuroanatomical and neurocircuitry factors.

### Theoretical Model of Panic Disorder Factors

#### Neuroanatomical Theory of PD

Gorman and colleagues proposed the neuroanatomical theory of PD that suggests the involvement of discoordination of neural circuitry and dysfunctional integration of information in both cortical and subcortical regions [6]. According to Gorman, whilst anticipatory anxiety involves the limbic structures and the prefrontal cortex (PFC) is responsible for phobic avoidance [9], PAs are a result of increased activity of the noradrenergic neurons of the locus coeruleus (LC). Psychopharmacological interventions such as selective serotonin reuptake inhibitors (SSRIs) were hypothesized to reduce PAs by decreasing the activity in the amygdala and by inhibiting projections to the brainstem and other subcortical sites [6]. Andrisano *et al*. [10], in their meta-analysis, demonstrated a close link between serotonin and PD and tryptophan depletion as evidenced by the increase of PAs and anxiety symptoms in PD. Moreover, higher anti-panic efficacy in SSRIs as compared with other medications was noted in their meta-analysis. Klein [11] suggested that serotonergic antidepressants are efficient in treating spontaneous and situationally predisposed PAs. To date, the mechanisms of how SSRIs work therapeutically remain unknown [12].

Conversely, psychotherapies including cognitive behavior therapy (CBT) decondition contextual fear, decrease cognitive misappraisals, and disproportionate emotional reactions. They achieve this by reinforcing and strengthening the ability of the medial PFC, and specifically the hippocampus, to inhibit the amygdala [6]. Based on similarities between conditioned fear responses in animals and PAs in humans, Gorman and colleagues revised their hypothesis to identify and map neuroanatomical pathways in humans [6]. Despite their panic amygdala model gaining popularity, it was later discredited by studies that found that patients devoid of the amygdala develop PAs spontaneously and in response to the 35% CO_2_ challenge [13]. Similarly, Wiest *et al*. [4] suggested that the initial pathology is not necessarily restricted to fear sites such as the amygdala, following the finding that a patient with bilateral selective lesions of the amygdala was experiencing PAs. Further support has been provided by numerous studies that demonstrated that the amygdala in humans with bilateral damage notably impairs the processing of fearful facial expressions [14, 15]. This contradicts previous findings that the amygdala is a key region in the initiation of PAs. On the contrary, several sites involved in fear circuitry have been implicated in the regulation of panic responses, including the prefrontal cortex, insula, thalamus, septohippocampal system, as well as the LC and raphe nuclei. Regulatory dysfunction at any of the abovementioned key sites in this fear network may lead to the initiation of PD symptoms [5].

## 2. Methods

This systematic review was conducted according to PRISMA guidelines. This review draws on articles found via the PubMed database. Clinical studies and a randomized controlled trial, published in English between 2010 and 2020, were selected. The keyword search included panic disorder*(neur*/fear circuitry/fear network/serotonin/amygdala/noradrenalin/biomarker/hypothalamus/corticotropin releasing* OR CRF OR CRH/functional near infrared spectroscopy OR fNIRS/angiotensin II type 1 receptor OR AT1R).

In the first step of the process, titles and abstracts were manually screened against the inclusion/exclusion criteria. At this stage, retained articles were assessed against the following inclusion criteria: (1) an original research paper, (2) focused specifically on PD with/without comorbidity, (3) focused on panic/fear circuitry, and (4) adult participants/animal studies. Articles are excluded if they were: (1) a meta-analysis/systematic review/theoretical literature, (2) unrelated to PD, (3) focused on the therapy modalities/pharmacological intervention of PD, and (4) without an abstract.

**Fig. 1.**
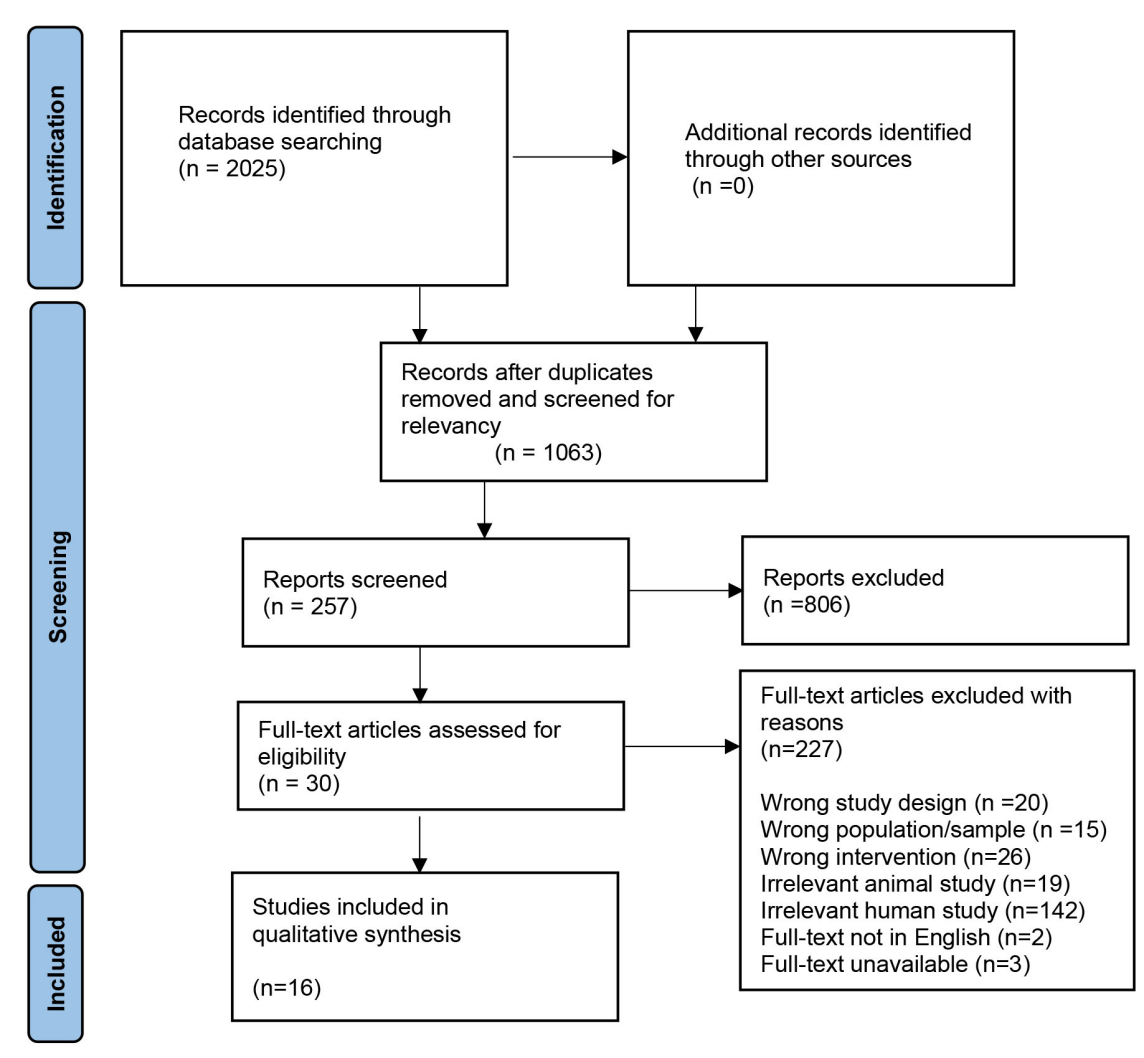
**PRISMA flow chart outlining the study identification and selection process**.

Next, full-text articles were screened for their eligibility for qualitative synthesis. The article inclusion process was conducted independently by two reviewers, PK and CW, with a third reviewer, RCF, involved to resolve any inclusion disagreement before proceeding. Satisfactory articles were included in the synthesis and quality assessment used (see Fig. 1 for the PRISMA flow chart outlining the study identification and selection process, and see Table 1, Ref. [16, 17, 18, 19, 20, 21, 22, 23, 24, 25, 26, 27, 28] and Table 2, Ref. [29, 30, 31] for quality assessments of human and animal studies, respectively). The Newcastle-Ottawa Scale [32] was used to assess the quality and risk of bias in human studies (see Table 3, Ref. [16, 17, 18, 19, 20, 21, 22, 23, 24, 25, 26, 27, 28]). For assessing animal studies, the Systematic Review Centre for Laboratory Animal Experimentation (SYRCLE) [32] risk of bias tool was used (See Table 4, Ref. [16, 17, 18, 19, 20, 21, 22, 23, 24, 25]). In the systematic review, means and standard deviations were extracted from the primary data sources of included studies. This extraction process involved a detailed examination of the methodology and results sections of each study to identify the reported means and standard deviations pertaining to the relevant outcomes.

**Table 1.** **Quality assessment of human studies**.

Study	Selection	Comparability	Outcome	Total
Pannekoek *et al*. 2013 [16]	5	1	3	9
Feldker *et al*. 2018 [17]	5	1	3	9
Fonzo *et al*. 2015 [18]	5	1	3	9
Burkhardt *et al*. 2019 [19]	4	1	2	7
Killgore *et al*. 2014 [20]	5	1	3	9
Lieberman *et al*. 2017 [21]	5	1	3	9
Marin *et al*. 2017 [22]	5	1	3	9
Balderston *et al*. 2017 [23]	5	1	3	9
Gorka *et al*. 2014 [24]	5	1	3	9
Tuescher *et al*. 2011 [25]	4	1	2	7
Depperman *et al*. 2014 [26]	5	1	3	9
Lambert *et al*. 2011 [27]	5	1	2	8
Klahn *et al*. 2017 [28]	5	1	3	9

**Table 2.** **Quality assessment of animal studies**.

Component of Experimental Design	Santos *et al*. 2013 [29]	D’amico *et al*. 2017 [30]	Casarotto *et al*. 2010 [31]
Sequence generation	Unclear risk	Unclear risk	Low risk
Baseline characteristics	Low risk	Low risk	Low risk
Allocation concealment	Unclear risk	Low risk	Low risk
Random housing	Low risk	Low risk	Low risk
Investigator blinding	High risk	High risk	High risk
Random outcome assessment	High risk	High risk	High risk
Blinding outcome	High risk	High risk	High risk
Incomplete data	Unclear risk	Unclear risk	Unclear risk
Selective reporting	Low risk	Low risk	Low risk
Ethical consideration	Low risk	Low risk	Low risk

**Table 3.** **The Summary of Findings of Neuroanatomical Studies**.

Study	Sample	Intervention	Measures	Notes
Pannekoek *et al*. (2013) [16]	n = 11 PD (1 M, 10 F)	Nil	3T fMRI - resting state	All subjects were recruited from the MRI study from the large-scale longitudinal multi-center cohort Netherlands Study of Depression and Anxiety (NESDA)
	n = 11 HC (1 M, 10 F)			
Fonzo *et al*. (2015) [18]	n = 15 GAD, mean age 33.93 years (±10.55)	Emotion face assessment task	3T fMRI - BOLD
	n = 15 PD, mean age 27.00 years (±7.00)		
	n = 14 SAD, mean age 25.43 years (±8.55)			
	n = 15 HC, mean age 30.00 years (±10.21)			
	Participated in fMRI			
	n = 44 of 59			
	n = 10 GAD			
	n = 12 PAD			
	n = 12 SAD			
	n = 10 HC			
Feldker *et al*. (2018) [17]	n = 26 PD (18–46 years)	Panic-related Picture Set Münster (PAPSM), comprising 50 panic-related and 50 neutral scenes	3T fMRI	Comparable for age, sex, and education. Native German speaker, right-handed, normal, or corrected to normal vision.
	n = 26 HC (19–32 years)			
	n = 13 primary PD diagnosis			
	n = 13 primary PD w/agoraphobia diagnosis			
	6 patients undergoing psychotherapy at the time of the study participation			
Burkhardt *et al*. (2019) [19]	n = 17 PD (n = 2 male)	Standardized disorder-related and neutral scripts	3T fMRI	
	n = 17 HC (n = 4 male)			
Killgore *et al*. (2014) [20]	n = 22 HC	Masked facial affect paradigm - exposed to a series of photographs from the Ekman standard set of images.	3T fMRI	Recruited from flyers and internet advertisements within the Boston Metropolitan area
	n = 15 SP	Face detection task		
	n = 14 PTSD			
	n = 14 PD			
	Groups did not sig differ in age, education, or gender composition.			
Marin *et al*. (2017) [22]	n = 21 HC, n = 10 female (47.6%), n = 11 male (52.4%), M = 25.8 years, SD = 4.8 years	2-day fear conditioning and extinction paradigm	3T fMRI	
	n = 61 AD, n = 36 female (59%), n = 25 male (41%), M = 30.4 years, SD = 11.5 years.	Electrical stimulation		
	HC younger and more educated			
Gorka *et al*. (2014) [24]	n = 13 PD with MDD	Aversiveness task = two within subject factors - predictable vs unpredictable, valence vs neutral	3T fMRI	Samples were recruited from a larger study on emotional processes.
	n = 9 MDD with no lifetime AD			
	n = 19 no diagnosis			Clinical diagnoses made using the SCID for DSM-IV.
Tuescher *et al*. (2011) [25]	n = 8 PD	Thread and safe condition of stimulus sham condition of electrodermal stimulation	3T fMRI
	n = 8 PTSD			
	n = 8 HC			
Deppermann *et al*. (2014) [26]	n = 44 PD (22 to randomized sham, 22 to verum rTMS group)	Verbal Fluency Task (phonological task, semantical task, control task)	fNIRS	Groups did not differ in gender, age, years of education, and handedness.
	n = 23 HC	rTMS - iTBS		PD with or without agoraphobia was diagnosed using SCID for DSM-IV.
Balderston *et al*. (2017) [23]	n = 63 participants	7.5% CO_2_ challenge	3T fMRI	
	28 female (mean age = 27 years, SD = 5.7 years)	NPU paradigm		
Lieberman *et al*. (2017) [21]	n = 42 (mean age = 25.26 years, SD = 7.60 years)	Baseline screening of semi structured interview and battery questions	EMG	Participants either: (1) had anxiety or depressive symptoms severe enough to warrant treatment (as assessed via trained clinicians) and consented to treatment with pharmacotherapy (selective serotonin reuptake inhibitors [SSRIs]) or cognitive behavioral therapy [CBT]) (i.e., patients) or (2) had no lifetime history of psychopathology (i.e., healthy controls)
	62% of whom were Caucasian, 12% were African-American, 21% were Asian, 2% were American Indian, and 2% reported ‘Other’. Of these individuals, 14% were Hispanic and 74% were female		3T fMRI using an 8-channel phased-array radio frequency head coil	
		NPU-threat startle task		
Lambert *et al*. (2011) [27]	n = 6 hypertension		ECG and mMSNA recording	Selective sampling (participants are drawn from previous studies)
	n = 6 major			
	depressive			
	n = 7 MDD			
	n = 9 PD			
Klahn *et al*. (2017) [28]	n = 22 PD (of which two dropped out due to anxiety before scanning)	NPU paradigm - fearful or neutral facial expression and scare video for predictable and unpredictable condition	MEG	
	n = 20 SP (both according to DSM-IV-TR-criteria)			
	n = 20 HC and 20 non-anxious controls (for detailed characteristics of the sample)			

Notes: fMRI, functional magnetic resonance imaging; BOLD, blood oxygenation level dependent; fNIRS, functional near-infrared spectroscopy; EMG, electromyography; ECG, electrocardiogram; MSNA, muscle sympathetic nerve activity; rTMS, repetitivetranscranial magnetic stimulation; iTBS, intermittent theta burst stimulation; NPU, no threat predictable unpredictable; MEG, magnetoencephalography; PD, panic disorder; HC, healthy controls; MDD, major depressive disorder; SP, social phobia; GAD, generalized anxiety disorder; PAD, panic disorder; SAD, social anxiety disorder; PTSD, post traumatic stress disorder; AD, anxiety disorder; SCID, structured clinical interview for DSM disorders, DSM-IV, diagnostic and statistical manual of mental disorders fourth edition, SD, standard deviation; F, female; M, male.

**Table 4.** **Summary of Brain Imaging Findings of Neuroanatomical Studies of PD and AD**.

Study		Scanner Type	Lateral-isation	MNI/Talairach coordinates				Main findings	*p*-value
				X	Y	Z			
Amygdala									
	Pannekoek *et al*. (2013) [16]	3T	R	–44	–66	38		PD showed increased negative connectivity in the right amygdala with the bilateral precentral and postcentral gyrus, the right supplementary motor cortex, and the rACC compared with HC	<0.05
	Fonzo *et al*. (2015) [18]	3T	R	20	–7	–13		AD groups have greater positive differential activation between processing fear and happy conditions relative to HC	0.006
	Feldker *et al*. (2018) [17]	3T	R	24	5	13	^∧^	PD patients showed significant hyperactivation (panic-related > neutral scenes) compared with HC	<0.05
			L	–17	–5	–14	^∧^	PD patients showed significant hyperactivation (panic-related > neutral scenes) compared with HC	<0.05
	Burkhardt *et al*. (2019) [19]	3T	R	25	–3	18	^∧^	PD patients showed higher amygdala activation in response to disorder-related vs natural scripts compared with HC	<0.05
	Lieberman *et al*. (2017) [21]	3T	R	32	–4	–12		Significantly activated in all participants in U-threat	<0.05
	Killgore *et al*. (2014) [20]	3T	L	–22	2	–24		All anxiety groups > HC for fear vs neutral contrast context	<0.001
			L	–22	2	–22		All anxiety groups > HC for happy vs neutral contrast context	<0.001
Hippocampus									
	Killgore *et al*. (2014) [20]	3T	R	38	–18	–16		PD > SP for fear vs happy contrast context	<0.001
	Marin *et al*. (2017) [22]	3T	R	32	–30	–6		HC > PD, greater activation during late conditioning	0.007
Parahippocampal gyrus									
	Fonzo *et al*. (2015) [18]	3T	R	36	–23	–8		Positive relationships between trait anxiety and brain activation	0.001
	Killgore *et al*. (2014) [20]	3T	R	20	–36	–14		PD > HC, significantly higher activation for fear vs neutral contrast context	<0.001
Thalamus									
	Feldker *et al*. (2018) [17]	3T	L	–15	–19	6	^∧^	PD patients showed significant hyperactivation (panic-related > neutral scenes) compared with HC	<0.05
	Balderston *et al*. (2017) [23]		L	–9	15	9		P > U, N, significantly more activity only in fear network	<0.001
vmPFC									
	Marin *et al*. (2017) [22]	3T	L	–8	50	–28		HC > AD, greater activation in early conditioning	0.009
		3T	L	–14	46	–18		AD < HC, less activation in extinction recall	0.02
	Burkhardt *et al*. (2019) [19]	3T	R	14	61	–4	^∧^	In PD, decreased activation during imagination of disorder-related vs neutral script	<0.05
	Killgore *et al*. (2014) [20]	3T	L	–12	40	–20		PD < HC, showed significantly decreased activation for fear vs natural contrast context	<0.001
	Balderston *et al*. (2017) [23]		B	3	–57	–6		P > N, U > N, significantly less activity to the predictable cue and unpredictable cue compared with the neutral cue in DMN	<0.001
vlPFC									
	Burkhardt *et al*. (2019) [19]	3T	R	22	55	5	^∧^	In PD, decreased activation during imagination of disorder-related vs neutral script	<0.05
		3T	L	–27	40	3	^∧^	In PD, decreased activation during imagination of disorder-related vs neutral script	<0.05
dmPFC									
	Burkhardt *et al*. (2019) [19]	3T	L	–4	62	11	^∧^	In PD, decreased activation during imagination of disorder-related vs neutral script	<0.05
	Balderston *et al*. (2017) [23]		B	3	3	51		P > U, N, significantly more activity only in fear network	<0.001
dlPFC									
	Burkhardt *et al*. (2019) [19]	3T	R	31	10	33	^∧^	In PD, decreased activation during imagination of disorder-related vs neutral script	<0.05
	Balderston *et al*. (2017) [23]		L	27	–24	51		P > U, N, significantly less activity for predictable cues compared with the unpredictable and neutral cues, and unpredictable cues compared with neutral cues in DMN	<0.001
Insula									
	Marin *et al*. (2017) [22]	3T	L	–36	10	–12		AD < HC, less activation in extinction recall	0.003
	Feldker *et al*. (2018) [17]	3T	L	–32	0	18	^∧^	PD patients showed significant hyperactivation (panic-related > neutral scenes) compared with HC	<0.05
		3T	L	–47	12	–13	^∧^	PD patients showed significant hyperactivation (panic-related > neutral scenes) compared with HC	<0.05
		3T	L	–38	–1	–7	^∧^	PD patients showed significant hyperactivation (panic-related > neutral scenes) compared with HC	<0.05
	Killgore *et al*. (2014) [20]	3T	R	34	–16	14		PD > SP for happy vs neutral contrast context	<0.001
	Lieberman *et al*. (2017) [21]	3T	R	50	12	–4		Significant activation across all participants in U-threat	<0.05
	Gorka *et al*. (2014) [24]	3T	L	–36	–2	18		PD-MDD group showed greater activation during the U-Negative	<0.05
			R	34	–20	20		PD-MDD group showed greater activation during the U-Negative	<0.05
	Balderston *et al*. (2017) [23]	3T	L	57	24	18		P > U, N, significantly more activity only in fear network	<0.001
			R	–51	–3	3		P > U, N, significantly more activity only in fear network	<0.001
			R	–63	36	21		P > U, N, significantly more activity only in fear network	<0.001
PCC									
	Burkhardt *et al*. (2019) [19]	3T	Dorsal	14	–44	38	^∧^	In PD, decreased activation during imagination of disorder-related vs neutral script	<0.05
	Balderston *et al*. (2017) [23]		B	0	60	24		P > U, N, significantly less activity for predictable cues compared with the unpredictable and neutral cues, and unpredictable cues compared with neutral cues in DMN	<0.001
MCC									
	Feldker *et al*. (2018) [17]	3T	L/R	–1	–1	30	^∧^	PD patients showed significant hyperactivation (panic-related > neutral scenes) compared with HC	<0.05
			L/R	–5	19	38	^∧^	PD patients showed significant hyperactivation (panic-related > neutral scenes) compared with HC	<0.05
ACC									
	Feldker *et al*. (2018) [17]	3T	L	–4	19	37	^∧^	PD patients showed significant hyperactivation (panic-related > neutral scenes) compared with HC	<0.05
			L	–3	39	17	^∧^	PD patients showed significant hyperactivation (panic-related > neutral scenes) compared with HC	<0.05
dACC									
	Pannekoek *et al*. (2013) [16]	3T	L	2	50	28		PD > HC, decreased connectivity with the bilateral frontal pole and superior/medial frontal gyrus	<0.05
			R	38	–32	48		PD > HC, increased left dACC connectivity with the bilateral pre-central and post-central gyrus	<0.05
	Lieberman *et al*. (2017) [21]	3T	R	2	16	42		Greater activation associated with greater panic symptoms (IDAS-II) during U-threat	<0.05
rACC									
	Marin *et al*. (2017) [22]	3T	L	–12	44	8		AD < HC, less activation in extinction recall	0.007
	Burkhardt *et al*. (2019) [19]	3T	L	–11	32	–7	^∧^	HC > PD activation during imagination of disorder-related vs neutral script	<0.05
		3T	R	7	31	–6	^∧^	In PD, decreased activation during imagination of disorder-related vs neutral script	<0.05
Subgenual cingulate									
	Tuescher *et al*. (2011) [25]	3T	R	6	12	–9		PD vs PTSD showed less activation bin the Threat vs Safe contrast	0.05
Dorsal midbrain									
	Tuescher *et al*. (2011) [25]	3T	R	6	–24	–18		Relative increase in the interaction contrast PD vs PTSD and Threat vs Safe	0.003
Brainstem									
	Feldker *et al*. (2018) [17]	3T	L	–4	–34	–15	^∧^	PD patients showed significant hyperactivation (panic-related > neutral scenes) compared with HC	<0.05
			L	–8	–32	–38	^∧^	PD patients showed significant hyperactivation (panic-related > neutral scenes) compared with HC	<0.05
Right caudate									
	Tuescher *et al*. (2011) [25]	3T	R	9	12	3		Relative increase in the interaction contrast PD vs PTSD and Threat vs Safe	0.045
S1									
	Balderston *et al*. (2017) [23]	3T	L	36	24	54		P > U, N, significantly more activity only in fear network	<0.001
IPS									
	Balderston *et al*. (2017) [23]	3T	L	39	78	36		P > U, N, significantly less activity for predictable cues compared with the unpredictable and neutral cues in DMN.	<0.001

^∧^ Talairach coordinates. PD, panic disorder; AD, anxiety disorders; SP, social phobia; HC, healthy controls; U-Threat, unpredictable threat; IDAS, Inventory for Depression and Anxiety Symptoms; DMN, default mode network; rACC, rostral anterior cingulate cortex; MNI, Montreal Neurological Institute; vmPFC, ventromedial prefrontal cortex; vlPFC, ventrolateral prefrontal cortex; dmPFC, dorsomedial prefrontal cortex; dlPFC, dorsolateral prefrontal cortex; PCC, posterior cingulate cortex; MCC, midcingulate cortex; ACC, anterior cingulate cortex; dACC, dorsal anterior cingulate cortex; rACC, rostral anterior cingulate cortex; IPS, left intraparietal sulcus; P, predictable; U, unpredictable; N, no threat.

## 3. Results

The keyword search generated a total of 2025 articles. Duplicates and irrelevant articles were removed, and 1063 articles were retained for abstract screening. The exclusion criteria removed 806 articles and 30 articles were included for the full-text assessment. In the full-text screening stage, 227 articles were excluded and 16 articles were included in the qualitative data synthesis. The 16 articles were analyzed, and findings were synthesized into one main category: neuroanatomical studies, with a primary focus on explaining PD etiology.

This paper reports solely on the neuroanatomical findings from this systematic review, which encompasses both human and animal studies.

### 3.1 Neuroanatomical Human Studies

The database search yielded 13 human studies meeting the inclusion criteria. Nine of the human studies compared the brain activity and/or connectivity between different brain regions in PD patients. The remaining studies investigated neurophysiological connections in the brain and the physiological effect of their intervention on PD. A summary of the neuroanatomical studies can be found in Table 3. Descriptive statistics for these studies include mean ± standard deviation (SD).

The brain imaging studies adopted a variety of interventions/exposure methods (i.e., fear/extinction conditioning, predictable and non-predictable cues [NPU paradigm], aversiveness task, emotional face observation task, and verbal fluency task [VFT]) as well as the absence of intervention (i.e., resting state functional connectivity [RSFC]). The non-brain imaging studies were observed to implement more CO_2_ challenge interventions to induce panic.

**Fig. 2.**
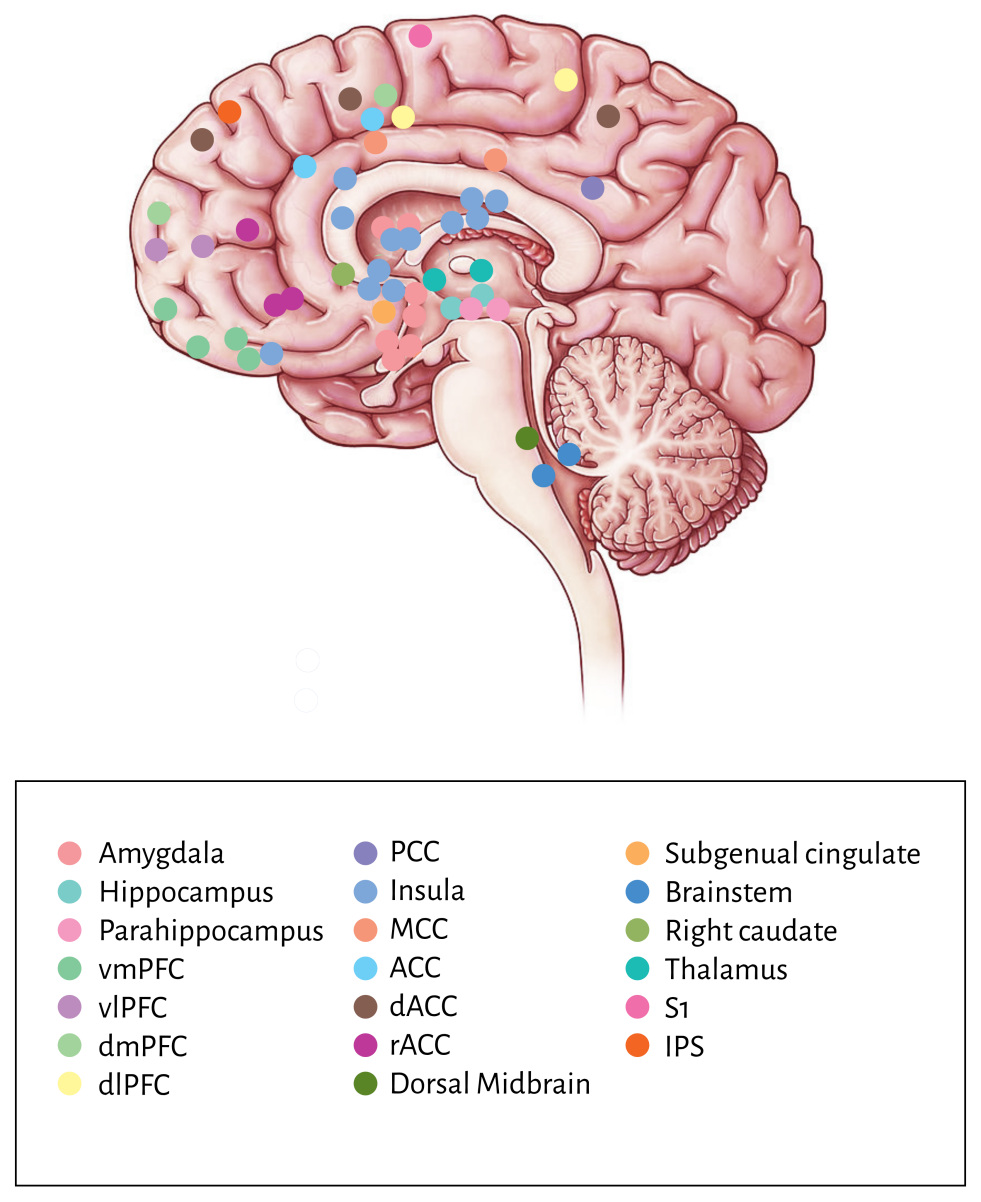
**Brain map illustration of the neural activation of the brain imaging study results**. Note: The brain map illustration is mapped against a standardized model of a lateral brain view. The colored dots were mapped according to the MNI/Talairach coordinates stated in Table 2. There may be some degree of deviation in the location of the colored dots. vmPFC, ventromedial prefrontal cortex; vlPFC, ventrolateral prefrontal cortex; dmPFC, dorsomedial prefrontal cortex; dlPFC, dorsolateral prefrontal cortex; PCC posterior cingulate cortex; MCC, midcingulate cortex; ACC, anterior cingulate cortex; dACC, dorsal anterior cingulate cortex; rACC, rostral anterior cingulate cortex; IPS, intraparietal sulcus.

The findings of the brain imaging studies are summarized in Table 4. Brain activation is noted in the Montreal Neurological Institute (MNI) coordinate system or the Talairach coordinates. Based on these coordinates, a map of brain activation was created to better illustrate the neurocircuitry of Parkinson’s disease patients undergoing various interventions (see Fig. 2).

#### 3.1.1 Amygdala

Neural activity in the amygdala in PD patients was highlighted in six studies. In this systematic review, only one neuroanatomical study within the perimeter of the database search adopted the resting state functional connectivity approach. Pannekoek *et al*. [16] studied the aberrant limbic and salience network resting-state functional connectivity in PD patients without comorbidity. The study implemented a seed-based correlation approach to investigate the RSFC in PD patients. Three seed regions using the MNI coordinates were observed that included the amygdala, dorsal anterior cingulate cortex (dACC), and posterior cingulate cortex (PCC). Based on this seed region, Pannekoek *et al*. [16] found that compared with healthy controls (HC), the amygdala has negative connectivity at coordinates with the following regions: bilateral precentral and postcentral gyrus, right supplementary motor cortex, and the rostral anterior cingulate cortex (rACC).

Meanwhile, four studies investigated the neural activity of PD patients using either panic, disorder-related, or fear-related scripts/scenes/contexts and demonstrated similar findings. Four studies noted PD patients experience significant hyperactivation in the amygdala region compared with their HC counterparts [17, 18, 19, 20]. Each study seems to show consistent results, indicating that PD patients exhibit increased activation in the amygdala in fear/panic/disorder-related scripts or contexts. Hyperactivation is noted in both the right amygdala [17, 18, 19, 21] and the left amygdala [17, 20].

#### 3.1.2 Hippocampus

A similar part of the hippocampus was noted for its higher activation in two studies. Killgore *et al*. [20], who studied brain activity in a fear vs happy context, identified the hippocampus as one of the regions with significantly higher activation in PD compared with patients with social phobia (SP) and HC in a fear vs happy contrast context. Hippocampus activity was also observed in a fear conditioning study by Marin *et al*. [22], which found that HC had higher hippocampus activity compared with PD in late conditioning.

#### 3.1.3 Parahippocampal Gyrus

Killgore *et al*. [20] found significantly greater activation in the parahippocampal gyrus in PD patients compared with HC (*p* < 0.001) for a fear vs neutral contrast context. Fonzo and colleagues [18] assessed for brain activity in an emotional face task context and found significant positive relationships between trait anxiety and brain activation (*p* = 0.001). Both studies implemented conditioning intervention and activated a brain area that is similar and comparable.

#### 3.1.4 Thalamus

Activities in the thalamus were mentioned in two studies. Feldker *et al*. [17] indicated that hyperactivation was distinct in PD patients for panic-related scenes compared with neutral scenes. This hyperactivation is significantly higher in PD patients compared with HC (*p* < 0.05). The activation was noted in the left hemisphere. Balderston *et al*. [23] noted hyperactivation in the left hemisphere in PD for predictable threat compared with unpredictable and neutral contexts in the fear network (*p* < 0.001).

#### 3.1.5 Prefrontal Cortices

Multiple researchers highlighted the involvement of regions of the prefrontal cortex, which included the ventromedial prefrontal cortex (vmPFC), ventrolateral prefrontal cortex (vlPFC), dorsomedial prefrontal cortex (dmPFC), and dorsolateral prefrontal cortex (dlPFC), in the neural activity of PD patients under a variety of stimulus conditions.

Regarding the vmPFC, Marin *et al*. [22] found greater activation in the left vmPFC in HC compared with anxiety-disordered patients (PD included) in the early conditioning paradigm (*p* < 0.009) and less activation in the left vmPFC in extinction recall (*p* < 0.02). Burkhardt *et al*. [19] identified significantly decreased activation in the right vmPFC in PD patients during the imagination phase of the disorder-related vs neutral script (*p* < 0.05). Balderston *et al*. [23] found significantly lower bilateral activation of the vmPFC toward predictable and unpredictable cues compared with neutral cues in PD patients (*p* < 0.001).

vlPFC neural deactivation was noted only by one study, Burkhardt *et al*. [19]. They found vlPFC deactivation in both the left and right vlPFC. The hypoactivation is more relevant for the phase of the related disorder than the neutral script imagination.

Similar hypoactivation in relation to the related disorder and neutral script imagination was noted in the left dmPFC [18]. Interestingly, PD patients experienced significantly higher activation of the dmPFC during a predictable cue compared with an unpredictable and neutral cue only in the fear network (*p* < 0.001) [23].

The same two studies, Burkhardt *et al*. [19] and Balderston *et al*. [23], highlighted similar neural responses of the dlPFC in PD patients. Burkhardt *et al*. [19] discovered that the right dlPFC showed decreased activation during disorder-related imagination compared with neutral script (*p* < 0.05). The left dlPFC is significantly less active for predictable cues vs neutral cues in the default mode network (DMN).

#### 3.1.6 Insula

Six studies identified activity in the insula at 11 coordinate points. As seen in Table 2, activity in the insula was observed in both the left and right hemispheres.

In the study by Marin *et al*. [22], anxiety disorder patients (PD patients included) showed higher activation in the insula during the extinction recall paradigm. Hyperactivation of the insula was also observed in a study by Feldker *et al*. [17] that utilized panic-related scene intervention where hyperactivation was noted at three points in the left insula. The three points of hyperactivation that were significantly activated for panic-related scenes vs neutral scenes compared with HC were noted. Interestingly, Killgore *et al*.’s study [20] that implemented a happy vs neutral context found that PD showed greater happy vs neutral contrast compared with HC.

In the NPU paradigm studies, Lieberman *et al*. [21] observed significant activation of the right insula for all participants in an unpredictable threat condition. More comprehensive results were found by Gorka *et al*. (2014) [24], whereby PD with Major Depressive Disorder (MDD) comorbidity patients showed greater left and right insula activation during unpredictable threat compared with MDD patients and HC. In addition, Balderston *et al*. [23] found that significantly higher insula activation was observed for predictable threat conditions in the fear network only in PD patients. Significant activities were noted in both hemispheres. The findings of these studies appear to strongly support that the insula has greater activation during an adverse related stimulus, i.e., unpredictable threat, predictable threat, and panic-related scenes.

#### 3.1.7 Cingulate Cortices

Amongst the other brain regions, five studies observed notably one or more activities in the regions of the cingulate cortices, which include: midcingulate cortex (MCC), anterior cingulate cortex (ACC), dorsal anterior cingulate cortex (dACC), and rostral anterior cingulate cortex (rACC).

Feldker *et al*. [17] found significant bilateral hyperactivation in the MCC region at two peak coordinates. The two points demonstrated hyperactivation, which was higher in panic-related scenes compared with neutral scenes of the intervention for PD patients than in HC.

Feldker *et al*. [17] also found that the ACC experienced significant hyperactivation for the same intervention phase as the MCC. Two coordinates in the left ACC demonstrated hyperactivation in PD patients during panic-related scenes as compared with neutral scenes.

Notable activity in the dACC was observed in two studies. Pannekoek *et al*. [16] discovered that at resting state, PD patients have decreased connectivity between the left dACC and the bilateral frontal pole and superior/medial frontal gyrus compared with HC. However, increased connectivity between the left dACC and the bilateral pre-central and post-central gyrus was noted. Lieberman *et al*. [21] found that greater activation in the dACC is associated with increased panic symptoms as measured with IDAS-II during U-threat.

For the rACC, two studies suggest similar findings that PD and AD experience less or lower activation in the rACC compared with HC. Marin *et al*. [22] identified that AD (including PD patients) show less activation in extinction recall. Meanwhile, Burkhardt *et al*. [19], found that HC has higher activation in the left rACC during imagination of disorder-related script compared with PD patients. Furthermore, the reduced activation in PD was noted particularly on the right rACC.

Lastly, the subgenual cingulate cortex was noted in one study. Tuescher *et al*. [25] identified that there is reduced activation in the subgenual cingulate cortex in response to threats and increased sensitivity of this region to safe conditions was reported in PD patients during an instructed fear-conditioning paradigm.

#### 3.1.8 Dorsal Midbrain

Only one study noted the activity of the dorsal midbrain for PD patients. Tuescher *et al*. [25] found a relative increase in the interaction contrast in PD patients compared with post-traumatic disorder (PTSD) patients in the threat vs safe condition paradigm.

#### 3.1.9 Brainstem

Finding for the brainstem region were like most findings in the cingulate cortices. The left brainstem was more hyperactivated in PD during panic-related than during neutral scenes compared with HC at two points.

#### 3.1.10 Right Caudate

Tuescher *et al*. [25] also identified activity in the right caudate where there was a relative increase of interaction contrast for PD vs PTSD and threat vs safe conditions.

#### 3.1.11 S1

Balderston *et al*. [23] found that S1 activity was significantly higher in response to predictable cues compared with unpredictable and neutral cues in PD patients, but this was observed only within the fear network.

#### 3.1.12 Intraparietal Sulcus

Balderston *et al*. [23], also found that the left intraparietal sulcus (IPS) showed significant hypoactivation for predictable cues compared with unpredictable and neutral cues for the default mode network (DMN).

### 3.2 Animal Studies

The database search yielded three animal studies that met inclusion criteria and investigated neuroanatomical areas. The results of the animal studies are reported in Table 5 (Ref. [29, 30, 33]) and include research on the neurocircuits and neurochemistry alterations that could be related to PD. Most animal studies evaluated the fear condition and its association with PD.

**Table 5.** **Summary of Animal Study Findings**.

Study	Objective	Subjects	Intervention	Translational findings
Neuroanatomical				
Santos *et al*., 2013 [29]	Evaluate TrkC in fear network brain regions.	TgNTRK3 mice	Shock fear conditioning paradigm, administration of ifenprodil, an NMDA receptor 2B antagonist or tiagabine, a GABA reuptake inhibitor and, 24 h later, contextual fear extinction. Water maze paradigm and novel object recognition test.	TrkC is highly expressed in the hippocampus, contributing to hippocampus hyperexcitability and aberrant fear circuit activation. The recovery of fear memory by tiagabine administered locally in the hippocampus might lead to new therapeutic options in PD.
D’Amico *et al*., 2017 [30]	Explore the role of NT3/TrkC system in contextual fear extinction.	TgNTRK3 mice	Shock fear conditioning paradigm, administration of NT3 and, 24 h later, contextual fear extinction.	NT3 induced synaptic plasticity in the modulation of pathological fear and thus identifies an entry site for the development of pharmacological support of cognitive behavioral therapy in PD.
Johnson *et al*., 2012 [33]	Use a 20% CO_2_-panic provocation model to screen orexin receptor antagonists alongside a benzodiazepine positive control for panicolytic properties.	Sprague-Dawley rats	After the exposure to hypercarbic and atmospheric air gases, rats were placed in the open field box for 5 min, then assessed in a social interaction test for 5 min.	ORX neurons in the DMH/PeF area are important for triggering coordinated panic reactions, and ORX1 receptor antagonists could be a novel therapy method for PD. ORX1 receptor antagonists reduce panic responses via neuronal networks involving the extended amygdala, periaqueductal gray, and medullary autonomic regions.

TrkC, Tropomyosin receptor kinase C.

Electrical and chemical activation of the dorsal periaqueductal grey (dPAG) in rats elicits fight and flight behaviors and cardiovascular changes. Because these responses are like those seen in people with PD, activation of this region has been proposed as an experimental model of PAs. In our review, research performing activation of the dPAG area assessed the effects of brain-derived neurotrophic factor (BDNF) and tyrosine receptor kinase B (TrkB) signaling in the PD model. Results demonstrated that BDNF panicolytic-like effects occur via γ-Aminobutyric acid type A (GABAA) -dependent mechanisms and that the tyrosine receptor kinase family is not only implicated in the dPAG area [31]. The receptors of this family are relevant for several CNS regions related to PD. Tropomyosin receptor kinase C (TrkC), for instance, plays a role in PD preclinical models by regulating hippocampus-dependent fear memories [29]. Furthermore, TrkC homeostasis is disrupted in the mPFC of TgNTRK3 mice and is crucial in fear extinction impairments. It was demonstrated that TrkC-induced synaptic plasticity in the control of pathological fear in a shock-fear conditioning paradigm [30].

The BLA-CeL circuit is necessary for fear memory acquisition and the retrieval of extinction memory.

A study that used a 20% CO_2_-panic provocation model in rats showed that orexin (ORX) neurons in the dorsomedial/perifornical regions are important for triggering coordinated panic reactions [33, 34]. In addition, ORX1 receptor antagonists reduce panic responses via neuronal networks involving the extended amygdala, periaqueductal gray, and medullary autonomic regions [33].

## 4. Discussion

This systematic review yielded findings related to the neuroanatomical factors playing a role in the etiology and pathophysiology of PD. A qualitative systematic review is best suited to highlight the most significant findings.

Neuroanatomical brain imaging findings in humans highlighted several key areas involved in the pathophysiology of Parkinson’s disease, including the amygdala, hippocampus, parahippocampal gyrus, thalamus, brainstem, prefrontal cortex (PFC), insula, and cingulate cortices. The cingulate cortices are comprised of the midcingulate cortex (MCC), the anterior cingulate cortex (ACC), the dorsal anterior cingulate cortex (dACC), and the rostral anterior cingulate cortex (rACC). The dorsal midbrain, right caudate [35], and left brainstem [20] are also implicated in a relative increase in interaction contrast in PD patients. Hypersensitivity in the brainstem and the amygdala play a role in the pathogenesis of PD and in the activation of the fear network which involves sub-cortical and cortical regions.

The amygdala was highlighted in six studies in this review, with four studies indicating that PD patients have significant hyperactivation in the amygdala region compared with HC. Moreover, the coordinates of the amygdala activity across the four conditioning studies indicate a substantial degree of overlap in both left and right lateralization [17, 19, 20, 21]. Overall, PD patients appear to have either hyperreactive or hypersensitive amygdala when stimulated with a non-neutral stimulus (i.e., fear contrast stimulus, happy contrast stimulus, angry contrast stimulus, panic-related scenes, disorder-related scripts). According to Pannekoek *et al*.’s study [16] the connectivity between the amygdala and the bilateral pre-central and post-central gyrus, the right supplementary motor cortex, and the rACC appear to be reduced in PD. Therefore, the connectivity related to emotional processing between the amygdala and the abovementioned linked brain region may be impaired in PD patients. The morphometric measurements of the amygdala may point to the pathophysiological mechanisms underlying PD [25]. The resilience in anxiety states such as PD might be inhibited by altered neuronal integration and validation of anxiety-related emotional stimuli [36]. Abnormalities in regulating emotional processing have also been noted to contribute to the pathophysiology of PD [37, 38].

Several neurotransmitters that have lower receptor binding in the amygdala, including GABAA and serotonin, have been reported. Particularly, a study that used a 20% CO_2_-panic provocation model in rats showed that orexin (ORX) neurons in the dorsomedial/perifornical regions are important for triggering coordinated panic reactions. Activation of ORX-synthesizing neurons induces a panic-prone state in the rat panic model [33]. ORX1 receptor antagonists reduce panic responses via neuronal networks involving the extended amygdala, periaqueductal gray, and medullary autonomic regions [33].

The hippocampus has also been implicated in fear circuitry, given its significant role in emotional regulation and contextualizing fear responses. Research has demonstrated that fear conditioning is compromised in patients with amygdala lesions; however, fear conditioning is not affected by hippocampal lesions [5, 39]. The hippocampus processes risk assessment, which is a fundamental aspect of emotional regulation aimed at appraising potential danger versus rewards [22]. Moreover, the role of the hippocampus in PD is in the expression of fear and anxiety elicited by learned fear contributing to the integration of defensive neural networks that make up the fear circuitry, comprising of the hippocampus, amygdala, nucleus accumbens, periaqueductal gray, ventromedial hypothalamus, thalamic nuclei, insular cortex, and several brain stem and prefrontal regions [22].

The hippocampus has been found to have higher activation in PD compared with patients with social phobia (SP) and HC in a fear vs happy contrast context [20] and higher hippocampus activity has been found in HC in comparison with PD patients in late conditioning [20, 22]. Killgore *et al*. [20] reported significantly greater activation in the parahippocampal gyrus in PD patients when compared with HC. Moreover, the left intraparietal sulcus showed significant hypoactivation for predictable cues compared with the unpredictable and neutral cues in PD patients for DMN. Although the S1 area showed significantly higher activity for predictable cues compared with unpredictable and neutral cues in PD patients, it was only in the fear network [23].

The human neuroanatomical brain imaging findings regarding PD might be a consequence of neurochemical alterations in the PD central nervous system, resulting in neuroimage alteration. This review described some common findings regarding the neurochemical factors involved in PD. For instance, the effects of brain-derived neurotrophic factor (BDNF) and tyrosine receptor kinase B (TrkB) signaling in the PD model were demonstrated to be an important site for dPAG activation, as BDNF panicolytic-like effects occur via γ-aminobutyric acid type A (GABAA)-dependent mechanisms. BDNF exerts a modulatory effect on the serotonergic system in brain loci (periaqueductal grey and dorsal raphe nucleus) [40]. The periaqueductal grey (PAG) has high levels of TrkB and BDNF receptor messenger RNAs and proteins [41].

In animal models, electrical or chemical stimulation of the PAG induces escape responses and autonomic changes that are like those observed in aversive situations [41, 42]. Using electrical stimulation of the dPAG as a model of panic [42, 43], intra-dPAG injections of serotonin (5-HT) [44], or GABA-enhancing drugs reduce the escape response triggered by this stimulation, suggesting a panicolytic-like effect [31]. Insights from animal studies suggest that GABAergic neurons can exert a strong inhibitory effect on the dorsomedial and posterior hypothalamic nuclei, thereby reducing the excitability of neurons involved in the development and expression of panic-like responses [45]. A specific hypothalamic nucleus, the dorsalmedial hypothalamic nucleus (DMH), and a dysfunction in its regulatory mechanism may be relevant in the genesis/maintenance of panic disorder [46].

Regarding other neurotransmitters, most of the brain lactate and glutamate concentrations change in PD patients. A significant difference in visual cortex lactate/N-acetylaspartate was observed in PD patients, during and following the visual stimulation and recovery period. An important finding also suggests that glutamatergic baseline concentration mainly determines the degree of glutamate + glutamine/creatine. Brain lactate in PD is argued to be influenced by excessive cerebral vasoconstriction that leads to brain hypoxia and metabolic disturbance [47]. In addition, investigation of the relationship between PD and serotonin reuptake inhibitors (SRIs) on the coupling of cortical and cardiac activity has found that PD patients have higher N300H magnitudes compared with HC. This phenomenon has been labeled ‘N300H’ to indicate a negative association between EEG amplitude at 300 ms and the heart period (the acceleration at subsequent beats) following an external stimulus. Moreover, SRI treatment resulted in greater N300H activity spread in PD patients than in non-SRI-treated PD patients.

### 4.1 Strengths and Limitations of this Study 

This review covers a wide range of topics related to PD pathophysiology and fills a knowledge gap in an area integrating human and animal neuroanatomical data regarding PD, using a systematic methodology. The data were not homogeneous enough to perform a meta-analysis, which would enrich the results, and there were too few articles on animal studies reviewed to adequately summarize the pathogenesis of PD. Therefore, more studies integrating human and animal neuroanatomical studies are required to better understand fear circuitry in the brain.

### 4.2 Implications for Research

Much of the research to date has focused on the dysregulation of central fear circuitry, including the limbic network, which involves connections between the amygdala, anterior cingulate cortex, and PAG during panic symptoms. The potential role of areas devoid of a blood-brain barrier in PD is important to investigate, especially given their connectivity to downstream sites responsible for the expression of behavioral and physiological responses.

Although animal studies have played a significant role in informing our understanding of the etiology, mechanisms, and fear circuitry involved in PD, much has yet to be determined regarding the neurobiological basis and pathophysiology of PD. Advanced translational models are called for to determine which animal research is of empirical value to humans and to further understand the molecular and neural systems involved in PD. Future directions must incorporate technological advances in neuroimaging techniques as well as additional human and animal research encompassing neuroanatomical, neurochemical, genetic, and epigenetic factors. These findings may guide the development of new treatments for PD patients, aiming to reduce the debilitating effects and overall burden of the condition.

## 5. Conclusions 

In this review, we have presented animal and human studies regarding the neuroanatomical areas that are salient in PD. These studies have identified patterns of altered expression in several biological systems, such as neurotransmission, the hypothalamic pituitary adrenal axis, and neuroplasticity, resulting in neuroanatomical modifications.

Complex emotional and cognitive processing in neuropsychiatric illnesses is associated with abnormal functioning of neural circuits, which incorporate several brain regions [22] that are responsible for varying types of defensive responses and fear circuitry. Therefore, an understanding of the brain regions involved and their functional connectivity may further inform our understanding of the neurobiological foundation of PD, further leading to the development of effective interventions [22].

## Registration and Protocol

This systematic review is registered under PROSPERO with registration number CRD42021247285. PROSPERO registration can be retrieved from https://www.crd.york.ac.uk/prospero/.

The PRISMA protocol was used for this systematic review and is described in the methods section of the article.

## Availability of Data and Materials

Data is available in the original research articles available via the PubMed database.

## Supplementary Material

Supplementary material associated with this article can be found, in the online version, at https://doi.org/10.31083/AP38756.
